# Development and nutritional and sensory evaluation of cachapinta (*Pseudoplatystoma* sp) pâté

**DOI:** 10.1002/fsn3.183

**Published:** 2014-11-13

**Authors:** Cátia Maria de Oliveira Lobo, Renata Torrezan, Ângela Aparecida Lemos de Furtado, Rosemar Antoniassi, Daniela De Grandi Castro Freitas, Sidinéa Cordeiro de Freitas, Ana Lúcia Penteado, Cássia Soares de Oliveira, Carlos Adam Conte Junior, Eliane Teixeira Mársico

**Affiliations:** 1 Universidade Federal Fluminense – UFFNiterói, RJ, Brazil; 2 Empresa Brasileira de Pesquisa Agropecuária – EMBRAPA-CTAARio de Janeiro, RJ, Brazil; 3 Universidade Federal do Rio de Janeiro – UFRJRio de Janeiro, RJ, Brazil

**Keywords:** Byproducts, Cachara, freshwater fish, Pintado, PUFA, sensory acceptance

## Abstract

This study developed a technique for the preparation of pâté from cachapinta (*Pseudoplatystoma* sp) waste. For this, frozen minced cachapinta fish was crushed in a mini cutter and homogenized with all other ingredients. The prepared pâté was stored in seamed and thermally treated cans (volume 170 g). Weight proportions of mean moisture, ash, protein, and lipid contents of the minced fish were 75.49, 1.00, 15.00, and 7.92 (g/100 g), respectively. The formulation of the developed pâté is in accordance with legislation for fish products. Cachapinta pâté is a product with high content of polyunsaturated fatty acids, low level of trans fat, and good indices of nutritional quality. Tests of sensory acceptance, purchase intent, and sensory attributes (except spreadability) averaged a score above 6.0, indicating acceptability of the product. Our study suggests that the potential of minced cachapinta for pâté production is high, and that it can contribute a value-added product to seafood consumption.

## Introduction

The production of high quality, protein-rich food by the fishing and aquaculture industries contributes importantly to the regional and national economy. However, in some Brazilian regions, extractive fishing is declining (with a trend toward stabilization) due to over-harvesting of natural stocks. In contrast, fish farming is increasing, capitalizing on water availability, favorable climate, and a wide range of Brazilian species appropriate for aquaculture (Bombardelli et al. 2005). In 2010, the national production of farmed fish reached 479,399 tons, representing a 15.3% increase over the production in 2009. Inland aquaculture contributes 82.3% of the total national production (Brazil 2011), supplying half of the total fish consumption in the world (Godoy et al. 2013).

The genus *Pseudoplatystoma* (Pimelodidae, Siluriformes) comprises the largest fish of the family, including the pintado (*Pseudoplatystoma corruscans*) and the cachara (*Pseudoplatystoma reticulatum*) that are found in the main South American hydrographic basins and are regionally known as “surubins” (Carvalho et al. 2007). In Brazil, surubins (*Pseudoplatystoma sp*) are of high commercial value because of their tasty meat with low fat content and a lack of intramuscular bones that give them high economic and social importance in the regions where they occur (Crepaldi 2008). The cachapinta is a hybrid of a female cachara and a male pintado and is cultured in several Brazilian fish farms because of its superior performance in productive systems compared to the pure species (Carvalho et al. 2007).

Fish per capita consumption in Brazil is disproportionately low compared to the growing production because this protein source is less common in metropolitan regions, reflected in the low diversity of most available fish products and their convenience. Fish consumption could be increased by providing products that combine nutritional quality and high convenience to meet the current consumer needs. A higher awareness of the benefits of healthy eating could increase the demand for a protein-rich diet with polyunsaturated fatty acids and low caloric value, provided by fish products (Bombardelli et al. 2005).

Regular consumption of fish is associated with a decrease of chronic diseases, because of its nutritional value: while it is low in saturated fats, it is a source of polyunsaturated fatty acids, high quality proteins of good digestibility, fat-soluble vitamins, especially calciferol and tocopherol, and minerals such as selenium, iodine, magnesium, and zinc (Health Canada 2009). In general, fish is marketed in natura, but this is gradually changing (Gonçalves 2011). According to FAO, 56% of the global fish production was processed, and 74% of which was designed for human consumption (FAO 2008). The nutritional value of solid waste from fish processing is high (protein-rich with fatty acids of the omega-3 series), thus encouraging technologies that allow its use in food production to increase the number of healthy and nutritional products (Feltes et al. 2010; Monteiro et al. 2012).

The average waste volume of the fish industry exceeds 50% of the production. This can cause severe environmental pollution (Boscolo 2001) if the waste is not properly used or disposed of. Fish waste processing is a key issue for the reduction of environmental damage, and provides additional income opportunities for industries that increase profitability and ensure sustainability (UCCI 2004; Monteiro et al. 2012). The capacity to manage fish waste is important for sustainable growth and socio-environmental responsibility of companies (Feltes et al. 2010).

Pâté is as a cooked, “ready to eat” product, popular in the gastronomy of varioumany countries with greatly appreciated sensory characteristics. While pâtés were traditionally prepared with goose “*foie-gras*” or pork liver, they are now increasingly based on fish due to its nutritional benefits, offering an increased variety of pâtés with various sensory characteristics (Minozzo et al. 2004). The objective of this study was to use cachapinta pulp, a waste product of the filleting industry, pâté preparation, and to assess microbiological quality, proximate composition, fatty acid profile, and sensory acceptability of the final product.

## Materials and Methods

### Raw material origin and condition

We acquired 5 kg of frozen cachapinta pulp from a company located in Mato Grosso do Sul. The pulp was transported to the Brazilian Agricultural Research Corporation (EMBRAPA-Food Agroindustry) in Rio de Janeiro where it was kept at −18°C ± 1°C until processing.

### Pâté processing

Dry ingredients (Table1) of the formulation were added after grinding the cachapinta pulp in a mini-cutter (Geiger, Pinhais, Brazil) with a double blade and two rotation speeds. The mass was homogenized before and after adding oil and water, until an emulsion was formed, and packaged in 170 g cans coated with epoxy varnish. For thermal treatment, the cans were seamed and autoclaved.

**Table 1:** Formulation of pâté prepared from cachapinta pulp

Ingredients	Percent
Cachapinta (*Pseudoplatystoma* sp) pulp	60.0
Sunflower oil	20.0
Filtered water	16.7
Spices	2.0
Additives	1.3
Total	100

Thermal treatment was carried out in a vertical, fixed vapor autoclave (Tecnofood, Pouso Alegre, Brazil). The temperature was monitored with copper alloy thermocouples attached to the geometric center of the can and connected to a TESTO ® (Testo AG-Lenzkirch, Baden-Wiirttemberg, Germany) recorder. One thermocouple was placed in a can and the other was used for monitoring the internal temperature of the autoclave. The binomial time × temperature used to ensure commercial sterility of the pâté was 115°C for 15 min, resulting in sterilization value (*F*_0_) of 6.7 min. The cans with pâté were stored at 25°C for 180 days.

### Microbiological analysis of the raw material

The *Staphylococcus coagulase positive* count (CFUg^−1^) was performed by plating the sample onto a Baird-Parker Agar plate, and incubation at 35°C for 48 h. The presence of colonies was confirmed by Gram stain, catalase, coagulase, and thermonuclease tests. *Salmonella sp* detection was performed on 25 g of the sample by preenrichment in lactose broth, incubated at 35°C for 24 h followed by selective enrichment in tetrathionate broth and Rappaport broth, and then for 24 h at 35°C and 42°C, respectively. The isolation of *Salmonella sp* was carried out on xylose lysine deoxycholate agar, hectoen agar, bismuth sulfite agar, and incubated at 35°C for 24–48 h. If *Salmonella* was isolated, identification was performed by a somatic serological test and the following determinations: TSI test, LIA test, Gram staining, urease test, methyl red and Voges–Proskauer test, malonate test, indol test, fermentation of dulcitol test, and the urease test (APHA 2001).

### Proximate composition

The proximate composition of cachapinta pulp and cachapinta pâté was determined according to the procedure of the Association of Official Analytical Chemists (AOAC 2000). The samples (*n* = 6) were analyzed for moisture content by drying at 105°C to a constant weight (∼6 h), for fixed mineral residue (ashes) by ignition in a muffle furnace at 550°C, and for total protein content by the Kjeldahl method, which involves the conversion of protein and organic nitrogen to ammonia during digestion with sulfuric acid in the presence of a mercury catalyst mixture. The acid digestion was made alkaline and the ammonia was distilled and titrated with standard acid. The percentage of nitrogen was determined and converted into protein by multiplying with a factor of 6.25. Crude fat content was determined by extracting lipids from dried samples using a solvent (petroleum ether), and subsequent weighing the extracted fat.

### Determination of fatty acids

Fatty acid methylation was carried out according to Hartman and Lago (1973). The methyl esters obtained were analyzed by gas chromatography in an Agilent 6890 (Agilent Technologies, Santa Clara, CA, EUA) chromatograph equipped with a flame ionization detector operated at 280°C, using a fused silica capillary column of cyanopropyl siloxane (60 m × 0.32 mm × 0.25 *μ*m) with the following temperature programming: initial temperature of 100°C for 3 min (isothermal); from 100°C to 150°C with a ramp of 50°C/min; from 150°C to 180°C with a ramp of 1°C/min; from 180°C to 200°C with a ramp of 25°C/min and kept at the final temperature of 200°C for 10 min. The injected volume was 1 *μ*L with an injection port temperature of 250°C, in split flow mode, at a 1:50 ratio. The identification was made by comparison of the retention times with the NU CHECK (Elysian, MN) standards nos. 62, 79, 87, and PUFA no. 3, PUFA no .1 from Supelco (Supelco, St Louis, MO, EUA). Quantification was performed by internal normalization.

### Nutritional quality indices of lipids

Atherogenicity index (AI) and thrombogenicity index (TI) were determined according to Ulbricht and Southgate (1991) and the ratio of hypo-/hypercholesterolemic fatty acids (HH) according to Santos-Silva, Bessa and Santos-Silva (2002). The performed calculations based on the FAME profile were:

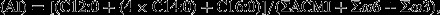


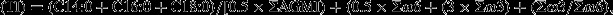


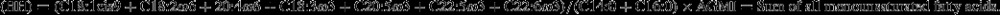


### Test of commercial sterility

Triplicate samples of cachapinta pâté were submitted to a commercial sterility test for low acidity foods (pH = 4.6). Patê cans were incubated, inverted in filter paper, in an oven at 36 ± 1°C for 10 days and at 55 ± 1°C for 5–7 days. After this, the integrity of the cans was verified according to the procedures described in Instruction 62 of the Ministry of Agriculture, Livestock and Supply (Brazil 2003).

### Acceptance test

Acceptance tests were carried out in individual booths of the sensory analysis laboratory (EMBRAPA Food Agroindustry) according to Meilgaard et al. (1991) and Hough et al. (2006). The cachapinta pâté was tested for acceptance on the 20th storage day by 96 untrained panelists and on the 180th storage day by 100 untrained panelists. Purchase intent was assessed based on the five-point hedonic scale, which ranges from 1 (will definitively buy) to 5 (will definitively not buy). The attributes “appearance,” “spreadability,” “flavor” and “overall acceptance” were assessed based on the nine-point hedonic scale, which ranges from 1 (dislike extremely) to 9 (like extremely). Each panelist received ∼30 g of pâté served at room temperature in 50 mL white disposable plastic cups coded with three random digits. The mean values obtained in the acceptance test were compared by the Student's *t*-test at a 5% significance level.

## Results and Discussion

### Analysis of raw materials

Fish pulp is a by-product with a sufficient content of proteins and lipids to be used as a base for novel food products of high value. Fish waste is a source of various nutrients, especially of omega-3 polyunsaturated fatty acids (n-3 PUFAs) (Stevanato et al. 2010). The protein content of cachapinta pulp in our study was similar to that of muscle tissue of cachara (*P. reticulatum*) and pintado (*P. corruscans*) fillets (Ramos Filho et al. 2008), and higher than of pulp from tilapia (*Oreochromis niloticus*) heads and carcasses (Monteiro et al. 2012). Cachapinta pulp contains a high amount of proteins and therefore can be recommended for human consumption.

Quality results of the raw material (cachapinta pulp) indicated adequate hygienic sanitary conditions due to the absence of *Salmonella* sp. in a 25 g sample and a positive coagulase *Staphylococcus* count below 1 log cfu g^−1^. The nutritional value of cachapinta pulp can be considered relevant, since significant amounts of protein and lipid were found (Table2).

**Table 2:** Mean proximate composition (G/100 g) ± SD of cachapinta pulp and cachapinta pâté

Sample *n* = 6	Moisture	Ashes	Protein	Lipids
Pulp	75.49 ± 1.5	1.00 ± 0.13	15.00 ± 0.20	7.92 ± 1.33
Pâté	63.14 ± 0.75	2.09 ± 0.18	8.34 ± 0.30	24.46 ± 0.16

### Evaluation of the efficiency of heat treatment

Since the canning process significantly increases the shelf life of fish products (Ogawa and Maia 1999), we performed a commercial sterility test and observed that the cans showed no evidence of leakage due to seam perforation or defect. Can swelling was not observed and were therefore approved for human consumption and sensory evaluation.

### Proximal composition of cachapinta pâté

The demand for food products with benefits beyond simple nourishment is increasing in modern society owing to a higher awareness of the health impacts of food (Basho and Bin 2010). Because consumers spend increasingly less time on food preparation, research institutes and food industries are developing nutritious products with added health benefits that are quick to prepare or ready-to-use (Gonçalves 2011; Monteiro et al. 2013). Pâté is a convenient and nutritious food. Fish pulp, potentially a waste product with high pollutant capacity, can be used as raw material for a novel fish product of high value and therefore has the potential to become a new income source for the fish industries.

The minimum standards for marketing pâté in Brazil are 70% maximum moisture, 32% maximum fat, 8% minimum protein, and 10% maximum for the sum of starch and total hydrocarbons (Brazil 2000). The mean proximate composition of cachapinta pâté meets these criteria (Table2). Protein and lipid content of our cachapinta pâté was similar to the creamy and pasty variants of tilapia pâté (*O. niloticus*), which contained 8.77% and 9.69% protein, 26.12%, and 28.15% lipid, respectively, achieved by high pulp content and the application of high temperatures (Minozzo et al. 2008). Current nutritional standards require healthier diets with higher fiber intake and lower saturated fat content. For this reason, fish is globally acknowledged as a food of high nutritional value that is beneficial to human health (Casotti 2002; Veloso 2005). Larsen et al. (2011) highlighted its nutritional benefits to human health, for example, by reducing cardiovascular diseases due to the presence of polyunsaturated fatty acids (n-3 PUFAS), in particular eicosapentaenoic acid (EPA; C20: 5n-3) and docosahexanoic acid (DHA;C22: 6n-3). Other seafood beneficial to human health include proteins, minerals, vitamins, and bioactive compounds such as taurine, phytosterols, antioxidants, and phospholipids. Among the minerals contained in seafood, the authors mention abundance of selenium, iodine, zinc, and calcium. Carvalho et al. (2006) link an increased selenium consumption to better protection against chronic diseases associated with aging such as atherosclerosis, cancer, arthritis, cirrhosis, and emphysema.

The fatty acid profile of cachapinta pâté shows a high content of unsaturated fatty acids (Table3), including the *ω*-3 and *ω*-6 families. There is a predominance of polyunsaturated fatty acids, especially linoleic acid (C18:2), which in addition to its presence in farmed fish like cachapinta, is contained in sunflower oil (between 64.6 and 71.5 g/100 g (Mandarino 2005), also used in pâté production. The concentration of monounsaturated fatty acids in cachapinta pâté was twice the concentration of saturated fatty acids. Highest concentrations were measured for palmitic acid (16:0) among saturated fatty acids; oleic acid (C18:1 *ω*9) among monounsaturated acids and linoleic acid (C 18:2 *ω*6) among polyunsaturated acids (Table3). The cachapinta pâté contained only a small amount of trans fatty acids (0.071 g/100 g) adding to its nutritional value.

**Table 3:** Fatty acid profile of cachapinta pâté

Fatty acid	g/100 g^1^	%^2^
C14:0	0.069	0.25
C16:0	2.485	9.07
C16:1 *trans*	0.034	0.12
C16:1 *cis*	0.216	0.79
C17:0	0.034	0.12
C18:0	1.271	4.62
C18:1 *cis*	8.344	30.33
C18:2 *trans*	0.037	0.13
C18:2 *cis ω*6	14.342	52.14
C20:0	0.075	0.27
C18:3 *ω*3	0.176	0.64
C20:1	0.092	0.33
C22:0	0.176	0.64
C20:4 *ω*6	0.064	0.23
C22:6 *ω*3	0.084	0.30
Σ AGS	4.110	14.97
Σ AGMS	8.652	31.45
Σ PUFA *ω*6	14.406	52.37
Σ *trans*	0.071	0.25
Σ PUFA *ω*3	0.260	0.94
Total lipids	28.747	–

1 g of fatty acid per 100 g sample.

2 percent of fatty acids in relation to total fatty acid content.

Unlike lipids from red meat with a high proportion of saturated fat, fish lipids are rich in polyunsaturated essential fatty acids of the omega series (Veloso 2005). The concentration of monounsaturated fatty acids in cachapinta pâté is similar to cachara fillets (Otani 2012) where the percentage of linoleic acid was highest in relation to total lipids. The similarity of the fatty acid profiles of cachara fillets and pâté prepared from cachapinta pulp indicates a predominance of theses acids in fish of the genus *Pseudoplatystoma* that live under similar conditions.

Several conditions influence the chemical composition of fish muscle. Sex, age, environment, season, and primary diet are reflected in the fatty acid profile (Bandarra et al. 2009). For example, the diet of farmed cachara includes sources of monounsaturated fatty acids such as oleic and linoleic acids to increase their concentration in the fish meat (Alasalvar et al. 2002).

### Analysis of nutritional quality index of pâté

Ulbricht and Southgate (1991) suggested the AI and the TI for evaluation of the quality of lipids in different food products. Saturated (lauric, myristic and palmitic) acids have the highest atherogenic potential. Of these, the capacity of myristic acid to increase cholesterol levels four times greater than that of lauric and palmitic acid. According to the TI, saturated fatty acids (myristic, palmitic and stearic) are considered prothrombogenic. The H/H ratio assesses the effects of fatty acids on cholesterol metabolism, and considers unsaturated fatty acids as hypocholesterolemic and saturated fatty acids (myristic and palmitic) as hypercholesterolemic (Santos-Filho et al. 2002). The nutritional quality assessment by AI, TI, and H/H of the pâté's lipid fraction yielded an AI of 0.12; a TI of 0.3, and an HH of 8.9.

### Sensory acceptance of pâté

Acceptance is a positive attitude that is measured by actual consumption of a food item, to evaluate how much an individual likes or dislikes a product (Gularte 2009). Sensory analysis is an indispensable tool when developing new products, or modifying existing products, for process optimization, cost reduction, shelf life determination, and market research (Queiroz and Treptow 2006).

Sensory acceptance tests did not reveal significant differences between the score means obtained on the 20th and the 180th day of storage (Student's *t*-test, *P* > 0.05) for either purchase intent or any other attribute except spreadability. Means were mostly above 6.0, indicating product acceptance (Table4). In comparison, sensory evaluation of creamy and pasty pâtés prepared with tilapia (*O. niloticus*) pulp yielded mean values of 7.40 and 6.40, respectively (Minozzo et al. 2008). Creamy pâté prepared with Nile tilapia (*O. niloticus)* had high sensory acceptance values in two regions of the state of Paraná (Minozzo and Waszczynskyj 2010). Pâtés prepared with mackerel meat and tuna liver also reached high acceptance indices in sensory evaluation tests (Aquerreta et al. 2002).

**Table 4:** Results of the sensory acceptance tests of cachapinta pâté during storage

Day	Consumers	Appearance	Spreadability	Flavor	Global acceptance	Purchase intent
20°	96	6.96	4.57*	7.21	6.78	3.74
180°	100	7.30	5.23*	7.54	7.08	4.05

* *P* < 0.05, the means are statistically different at a 5% probability.

## Conclusion

The cachapinta pâté prepared in this study proved to be a safe product for human consumption, with significant nutritional value and sensory acceptance. Therefore, with regard to innovation and sustainability, there is great potential in the use of cachapinta pulp for pâté production and hence in converting food industrial waste into an alternative seafood product of high value.

## Supporting Information

**Table S1.** Development and nutritional and sensory evaluation of cachapinta (*Pseudoplatystoma* sp) pâté.
