# Supplementary material for: Development and nutritional and sensory evaluation of cachapinta (Pseudoplatystoma sp) pâté
Source: Food Sci Nutr. 2014 Nov 13;3(1):10–6. doi: 10.1002/fsn3.183 (PMC4304557; doi:10.1002/fsn3.183)
Supplement: Supplementary file 1 — Table S1. Development and nutritional and sensory evaluation of cachapinta (Pseudoplatystoma sp) pâté. [file fsn30003-0010-sd1.ppt]

## Slide 1
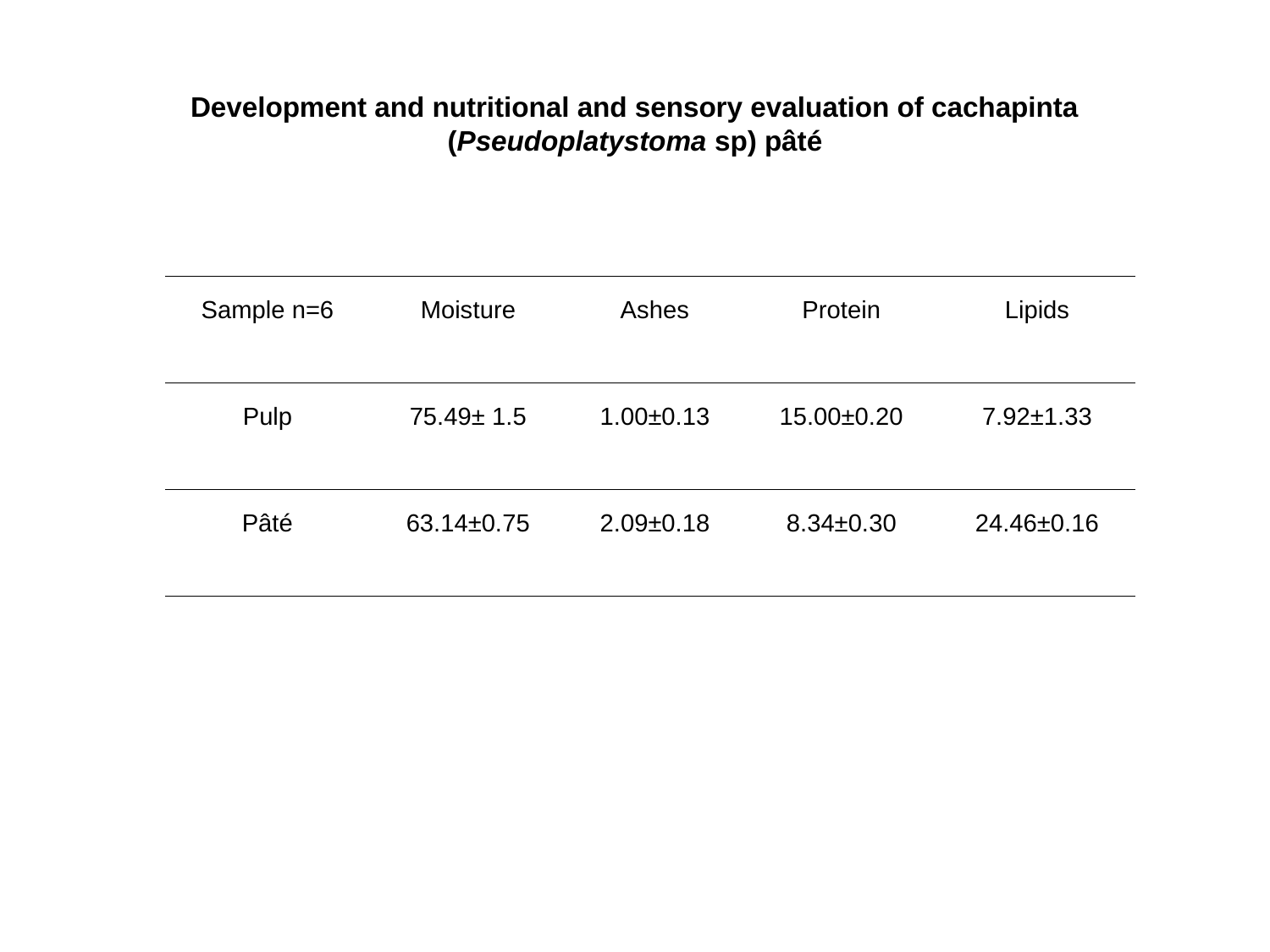

# Development and nutritional and sensory evaluation of cachapinta (Pseudoplatystoma sp) pâté
| Sample n=6 | Moisture | Ashes | Protein | Lipids |
| --- | --- | --- | --- | --- |
| Pulp | 75.49± 1.5 | 1.00±0.13 | 15.00±0.20 | 7.92±1.33 |
| Pâté | 63.14±0.75 | 2.09±0.18 | 8.34±0.30 | 24.46±0.16 |
